# Draft Genome Sequence of the Urinary Catheter Isolate *Enterobacter ludwigii* CEB04 with High Biofilm Forming Capacity

**DOI:** 10.3390/microorganisms8040522

**Published:** 2020-04-05

**Authors:** Sulman Shafeeq, Xiaoda Wang, Heinrich Lünsdorf, Annelie Brauner, Ute Römling

**Affiliations:** 1 Department of Microbiology, Tumor and Cell Biology, Karolinska Institutet, SE-171 65 Stockholm, Sweden; sulman.shafeeq@ki.se (S.S.); wangxiaoda@hotmail.com (X.W.); Annelie.Brauner@ki.se (A.B.); 2 Helmholtz Center for Infection Research, DE-38124 Braunschweig, Germany; hlunsdorf@web.de; 3 Clinical Microbiology, Karolinska University Hospital, SE-171 76 Stockholm, Sweden

**Keywords:** *Enterobacter ludwigii*, biofilm formation, genome sequencing, cyclic di-GMP, urinary catheter isolate, antimicrobial resistance

## Abstract

*Enterobacter ludwigii* is a fermentative Gram-negative environmental species and accidental human pathogen that belongs to the *Enterobacter cloacae* complex with the general characteristics of the genus Enterobacter. The clinical isolate *E. ludwigii* CEB04 was derived from a urinary tract catheter of an individual not suffering from catheter-associated urinary tract infection. The draft genome sequence of the high biofilm forming *E. ludwigii* CEB04 was determined by PacBio sequencing. The chromosome of *E. ludwigii* CEB04 is comprised of one contig of 4,892,375 bps containing 4596 predicted protein-coding genes and 120 noncoding RNAs. *E. ludwigii* CEB04 harbors several antimicrobial resistance markers and has an extended cyclic-di-GMP signaling network compared to *Escherichia coli* K-12.

## 1. Introduction

Urinary catheters are used worldwide in hospitals, health care units and community care, but increase the risk of catheter-associated urinary tract infections (CAUTIs) [1,2] with four out of five UTIs due to catheter usage [3,4]. A biofilm constitutes a microbial community which displays as cell aggregates or as adherent to a surface and/or interface enclosed by a self-produced or environment-derived extracellular matrix [5]. With an up to 10% risk of catheter surface colonization by microorganisms per day, detachment of pathogenic microorganisms from the catheter biofilm can cause CAUTI [6]. In addition, the catheter biofilm can serve as a reservoir for antimicrobial resistant microorganisms and can promote horizontal gene transfer of antimicrobial resistance genes [7].

Members of the *Enterobacter cloacae* complex are commonly isolated from urinary catheters [8,9,10]. The *E. cloacae* complex consisted originally of six Gram-negative species: *Enterobacter asburiae*, *E. cloacae*, *Enterobacter hormaechei*, *Enterobacter kobei*, *E. ludwigii* and *Enterobacter nimipressuralis* (now reclassified as *Lelliottia nimipressuralis* [11]). *E. ludwigii* is a fermentative, motile, rod-shaped bacterium first isolated from a clinical sample and established as a new species in 2005 [12]. Growth on 3-O-methyl-d-glucopyranose and myo-inositol differentiates *E. ludwigii* from other *Enterobacter* species [12]. As a versatile predominantly environmental species *E. ludwigii* isolates have been recognized as prominent electrogenic bacteria, as biofilm-forming heavy-metal-adapted isolates and as abundantly present in endophytic bacterial communities [13,14,15]. Furthermore, *E. ludwigii* isolates were characterized as bioremediation agents, alternating plant defense and capable of performing other important environmental functions [16,17,18]. In the clinical context, *E. ludwigii* is abundantly present in primary liver cancer [19]. This study reports the draft genome sequence and initial biofilm and antibiotic resistance characteristics of *E. ludwigii* CEB04 isolated from one patient not suffering from CAUTI. 

## 2. Materials and Methods

### 2.1. Growth Conditions and DNA Isolation

Luria–Bertani (LB) medium was used to grow *E. ludwigii* CEB04. To isolate genomic DNA, *E. ludwigii* CEB04 was grown in 50 mL LB medium overnight at 37 °C with shaking at 200 rpm. The genomic DNA of *E. ludwigii* CEB04 was isolated by Genomic-tip 500/G columns (QIAGEN, Hilden, Germany) and Genomic DNA buffer set (QIAGEN, Hilden, Germany) according to the manufacturer’s instructions. 

### 2.2. Genome Sequencing, Assembly and Annotation

The genomic DNA of *E. ludwigii* CEB04 was sequenced at the National Genomics Infrastructure (NGI, Science for Life Laboratory, Uppsala, Sweden) with PacBio RSII system (Pacific Biosciences, Menlo Park, CA, USA). *De novo* genome assembly was performed by HGAP4 (Hierarchical Genome-Assembly Process) algorithm from the PacBio SMRT tools [20]. The genome assembly was first polished with Quiver and a second polishing was performed using Arrow. Annotation of the genome was performed by the NCBI Prokaryotic Genome Annotation Pipeline (PGAP) [21] and the Rapid Annotations using Subsystems Technology (RAST; version 2.0) server [22,23,24]. 

### 2.3. Phenotypic Analysis

To visualize the biofilm phenotypes, bacteria were grown on LB without salt agar plates containing the dye Congo red (Sigma-Aldrich, Darmstadt, Germany) (40 µg/mL) and Coomassie brilliant blue G-250 (Sigma-Aldrich, Darmstadt, Germany) (20 µg/mL) incubated at 28 and 37 °C. Cell aggregation and pellicle formation were assessed visually after 24 and 48 h, respectively, with cells grown in LB without salt medium in standing culture at 28 and 37 °C. Swimming motility was performed at 37 °C in 0.3% LB agar with the swimming diameter measured after 6 h. Swarming motility was observed in 0.5% Eiken agar with 8% nutrient broth at 37 °C with the swarming diameter measured after 16 h. Control experiments were included as previously described [25,26].

To visualize *E. ludwigii* CEB04 biofilm formation on the catheter surface, a part of the catheter was incubated in human urine placed in a 96 well plate and incubated at 37 °C for 24 h. The catheter was washed with PBS and 1% glutardialdehyde was added for 24 h. The catheter was washed again with PBS pH 7.0, dehydrated in an acetone series and critically point-dried. After gold sputter coating, samples were analyzed with a scanning electron microscope (SEM; (Zeiss, Gemini 982, Oberkochen, Germany)) at 5 kV acceleration voltage at 9 mm width.

### 2.4. Genome and Protein Analysis

The average nucleotide identity (ANI) of *E. ludwigii* CEB04 to the EN119 type strain [12] was calculated as described previously [27]. NCBI conserved domain [28] and Prosite [29] databases were used to scan for GGDEF, EAL and HD-GYP domains in *E. ludwigii* CEB04. Paired protein alignment was conducted with Clustal Omega in Uniprot (www.uniprot.org/align) using standard parameters. Multiple sequence alignment was performed by MUSCLE, while MEGA 7.0 was used to create ML (Maximum Likelihood) phylogenetic trees [30]. Bootstrap analysis was performed for 1000 replicates. The genome was screened for antimicrobial resistance genes by ResFinder 2.1 [31]. 

## 3. Results

### 3.1. Biofilm Characteristics 

**Figure 1:**
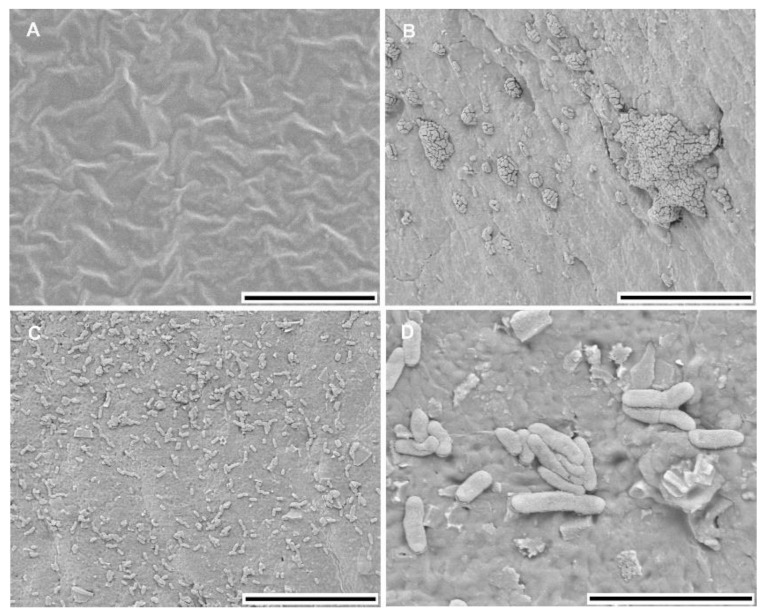
Scanning electron microscopy images showing biofilm formation of the urinary catheter isolate *E. ludwigii* CEB04 on the surface of a silicon catheter. Catheter control (**A**), different catheter areas with representative biofilm formation (**B**,**C**) and a detailed view of surface-attached cells (**D**). Scale bars = 20 µm (**A**–**C**) and 5 µm (**D**).

**Figure 2:**
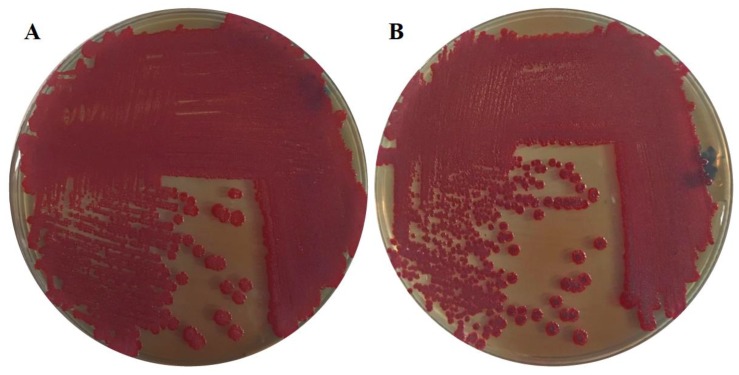
*E. ludwigii* urinary catheter isolate CEB04 grown on LB without salt agar plates containing Congo red and Coomassie brilliant blue. Plates were incubated for 48 h at 28 °C (**A**) and 37 °C (**B**).

The urinary catheter isolate *E. ludwigii* CEB04 displayed a high biofilm forming capacity on a silicon catheter surface (Figure 1) and exhibited various biofilm characteristics such as adherence to an abiotic polystyrene surface at 28 and 37 °C (Figure 1 and Figure 2 and Table 1). On a Congo red agar plate, *E. ludwigii* CEB04 showed the so-called pdar (pink, dry and rough) and rdar (red, dry and rough) morphotypes at 28 and 37 °C, respectively. This type of biofilm is characterized by the production of the extracellular matrix components cellulose (pdar) and curli fimbriae, which constitute the visible rdar morphotype [25]. Furthermore, *E. ludwigii* CEB04 had the ability to form a pellicle at 28 °C and exhibited cell aggregation in liquid culture at 37 °C. *E. ludwigii* CEB04 was non-haemolytic and showed swimming and swarming motility (0.2 and 3.2 cm, respectively) under standard conditions. 

### 3.2. Genome Assembly and Annotation

The sequence assembly resulted in a single contig of 4,892,375 bps representing the chromosome with a GC content of 54.5 %. Average Nucleotide Identity (ANI) indicated 98.98% identity compared to the genome of the *E. ludwigii* EN119 type strain [12]. MALDI-TOF mass spectrometry also identified strain CEB04 to belong to the species *E. ludwigii*. Annotation of the genome resulted in 4596 predicted protein-coding genes and 120 noncoding RNAs. Of note, subsequent Illumina sequencing is required to address inherent PacBio sequencing errors. Furthermore, PacBio sequencing might not have captured plasmids smaller than 10 kbp.

### 3.3. Antimicrobial Resistance Genes

The genome of *E. ludwigii* CEB04 was screened for antimicrobial resistance genes by ResFinder 2.1 [31]. This analysis indicated the presence of β-lactam, fosfomycin [32] and fluoroquinolone [33] resistance genes on the chromosome (Table 2). The antimicrobial resistance profile of *E. ludwigii* CEB04 showed resistance to ampicillin, fosfomycin, ciprofloxacin, cefadroxil, mecillinam and trimethoprim (data not shown), which extends the antimicrobial resistance profile obtained by in silico analysis.

### 3.4. GGDEF/EAL/HD-GYP Domain Proteins

Cyclic di-GMP is a second messenger promoting biofilm formation [26,34,35]. GGDEF domains are diguanylate cyclases, while EAL and HD-GHP domain proteins are c-di-GMP phosphodiesterases. We identified 15 GGDEF, 12 EAL, 10 GGDEF-EAL and 1 HD-GYP domain proteins in *E. ludwigii* CEB04, some of which have the same domain structure over the entire length of the proteins (Table 3). Nine proteins possess the RXXD motif indicative for a product-binding inhibitory (I)-site. Eight of these proteins are GGDEF domain proteins (EL-577, EL-703, EL-842, EL-1065, EL-1543, EL-1647, EL-3812 and EL-4124), while one is a GGDEF-EAL domain protein (EL-1102). Eight GGDEF, 8 EAL and 6 GGDEF-EAL domain proteins have homologues in *E. coli* K-12 MG1655. Closest homologues of all GGDEF/EAL/HD-GYP domain proteins are present in species belonging to the family of Enterobacteriaceae or to alpha-proteobacteria.

**Figure 3:**
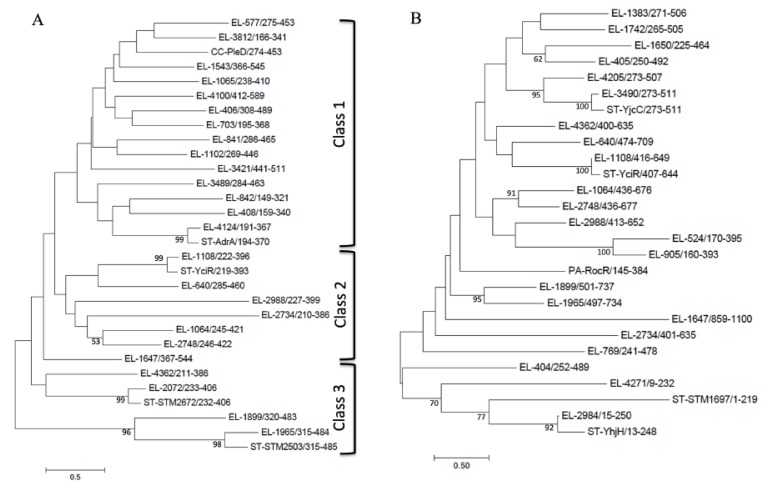
Phylogenetic trees of GGDEF domains (**A**) and EAL domains (**B**) in *E. ludwigii* CEB04. Numbers indicate amino acids of the protein included in the alignment. Bar indicates number of substitutions per site. EL= *E. ludwigii*, ST= *Salmonella typhimurium,* CC= *Caulobacter crescentus* and PA= *Pseudomonas aeruginosa*.

Alignment and subsequent phylogenetic analysis (Figure 3A and Supplementary data: Figure S1) clearly further classified the GGDEF domains from *E. ludwigii* CEB04 into three main classes according to their degree of sequence similarity, which is grossly reflected by the domain structure and, partially, the predicted functionality of the proteins. These three classes were enzymatically functional GGDEF domain proteins (class 1), enzymatically functional GGDEF domains linked to an EAL domain (class 2) and enzymatically non-functional GGDEF domains (class 3). Experimentally well-characterized GGDEF domain sequences from each GGDEF class were included for reference, such as the GGDEF domain of the diguanylate cyclase PleD [36] and AdrA [26] for class 1; YciR [37] for class 2; and STM2503 [37] for class 3. However, the classification according to domain structure does not robustly predict catalytic activity, as class 2 GGDEF-EAL proteins EL-2734 and EL-2988 have a degenerated GG(D/E)EF signature motif suggesting rapid evolution within each class [38]. Three GGDEF domain proteins (the two GAF-GGDEF proteins EL-408 and EL-842; two MASE4-GGDEF proteins EL-577 and EL-1102; and two dCache1-GGDEF proteins EL-406 and EL-3421,) and two MASE1-xGGDEF-EAL proteins (EL-1899 and EL-1965) have the same domain structure, indicating horizontal gene transfer of one of the copies or gene duplication (Figure 3A).

Alignment of the EAL domains and assessment of their phylogenetic relationship by a ML phylogenetic tree (Figure 3B and Supplementary data: Figure S2) showed a phylogenetic classification according to domain structure not to be as robust as previously documented for EAL domains of selected enterobacterial species [39]. However, EAL only domain proteins (EL-2984 and EL-4271), belonging to functional class IIa (enzymatically functional, but unconventional signature motifs) and class IIIb (non-enzymatic), cluster together as previously observed [38,39]. On the other hand, six redox-sensing CSS-EAL proteins (EL-404, EL-405, EL-1383, EL-1742, EL-3490 and EL-4205) and two light sensing BLUF-EAL proteins (EL-524 and EL-905) show homology over the entire length of the protein, however, their cyclic di-GMP turnover domains do not necessarily cluster in the phylogenetic tree (Figure 3).

Among the six CSS-EAL proteins, the EAL domain of the CSS-EAL protein EL-404 is most distant to the EAL domains of other CSS-EAL proteins suggesting recent horizontal transfer or domain shuffling. On the other hand, while clustering of the EAL domains of EL-1650 and EL-405 are supported by bootstrap values, the N-terminal signaling domain of EL-1650 is not a CSS domain [40] with the two cysteine residues in the putative periplasmic domain located in non-homologous positions. The two MASE4-GGDEF proteins have a low overall amino acid identity of 27.7%, the two GAF-GGDEF proteins a 24.3% identity, the two dCache1-GGDEF proteins a 20.3% identity and the two MASE1-GGDEF-EAL proteins have an identity of 29.4% over the entire length of the proteins, suggesting significantly different functionality and/or horizontal transfer from a distantly related species (xenologous genes). On the other hand, an identity of 44.1% of the two BLUF-EAL proteins indicates gene duplication and functional diversification (paralogous genes). To which extent gene duplication, horizontal gene transfer, domain shuffling and sequence evolution contribute to cyclic di-GMP turnover protein diversity in *E. ludwigii* remains to be investigated in future studies.

Structural biofilm genes required for expression of the rdar biofilm morphotype such as the curli biosynthesis operons *csgABC* and *csgDEFG* and the cellulose biosynthesis operons *bcsABZC* and *bcsEFG* are encoded on the chromosome of *E. ludwigii* CEB04 (data not shown).

## 4. Conclusions

Here, we report the whole genome sequence of *E. ludwigii* CEB04 isolated from the biofilm of a urinary tract catheter of one patient not suffering from CAUTI. This study is an initial investigation of the strain’s biofilm formation capability and identification of genes involved in biofilm formation, cyclic di-nucleotide second messenger signaling and antimicrobial resistance. More detailed investigations of these genes in *E. ludwigii* CEB04 will be required in order to analyze their distinct contribution to biofilm formation and antimicrobial resistance.

## Figures and Tables

**Table 1:** Biofilm characteristics of *E. ludwigii* CEB04.

	Pellicle ^1^	Cell Aggregation ^1^	Adherence ^1^	Morphotype ^1^
28 °C	+++	-	+++	pdar
37 °C	-	+	+	rdar

^1^ indicates no (-), low (+) and highly pronounced (+++) phenotypes.

**Table 2:** List of antimicrobial resistance genes found in *E. ludwigii* CEB04.

Resistance Gene	% Gene Identity ^1^	Chromosome Position	Predicted Phenotype	Accession Number of Reference
*blaACT-12*	99.21	3677688–3678833	Beta-lactam resistance AmpC-type	JX440355
*fosA2*	97.42	3879166–3879591	Fosfomycin resistance	EU487198
*oqxA*	86.82	4473130–4474305	Quinolone resistance	EU370913
*oqxB*	89.39	4474329–4477429	Quinolone resistance	EU370913

^1^ compared to reference over the entire length of the gene.

**Table 3:** List of GGDEF, EAL and HD-GYP domain proteins in *E. ludwigii* CEB04.

**Protein *E. ludwigii* CEB04**	**Locus Tag ^1^**	**Protein *E. coli* MG1655 ^2^**	**% Identity ^2,3^**	**Domain Structure**	**Highest Identity Outside the *Enterobacter* Genus**
**GGDEF domain proteins**					
EL-406	E5283_02015	-	-	dCache-1-GGDEF	*Kosakonia* sp.; 50.61%; WP043952574.1
EL-408	E5283_02035	YeaP	67.94	GAF-GGDEF	*Lelliottia nimipressuralis*; 86.26%; WP_134350745.1
EL-577	E5283_02875	YcdT	30.04	MASE4-GGDEF	*Leclercia* sp.; 73.95%; WP103793192.1
EL-703	E5283_03510	-	-	MASE5-GGDEF	*Lelliottia* sp.; 65.49%; WP103946328.1
EL-841	E5283_04195	-	-	Unknown-GGDEF	*Lelliottia* sp.; 82.52%; WP129034904.1
EL-842	E5283_04200	YeaP	32.52	GAF-GGDEF	*Lelliottia* sp.; 70.09%; WP064326108.1
EL-1065	E5283_05285	YdaM	68.54	PAS-PAS-GGDEF	*L. nimipressuralis*; 87.07%; WP_134350494.1
EL-1102	E5283_05465	YcdT	43.85	MASE4-GGDEF	*Klebsiella oxytoca*; 78.12%; SAP54703.1
EL-1543	E5283_07695	YdeQ	62.15	CHASE7-xCache-GGDEF	*K. oxytoca*; 78.12%; SAP54703.1
EL-2072	E5283_10410	YfiN	66.17	CHASE8-HAMP-GGDEF	*L. nimipressuralis*; 93.84%; WP_134347265.1
EL-3421	E5283_17410	-	-	dCache_1-GGDEF	*L. nimipressuralis*; 85.22%; WP_134348056.1
EL-3489	E5283_17760	-	-	Unknown-PAS-GGDEF	*Lelliottia* sp.; 73.66%; WP123429896.1
EL-3812	E5283_19415	-	-	Unknown-GGDEF	*L. nimipressuralis*; 93.84%; WP_134347265.1
EL-4100	E5283_20885	-	-	TMAO_torS-HAMP-NarQ-GGDEF	*Citrobacter* sp.; 79.97%; MTZ80699.1
EL-4124	E5283_21005	AdrA	61.31	MASE2-GGDEF	*Lelliottia* sp.; 76.13%; WP103948306.1
**EAL domain proteins**					
EL-404	E5283_02005	-	-	CSS-EAL	*Klebsiella* sp.; 79.66%; SSW82448.1
EL-405	E5283_02010	YcgG	50.40	CSS-EAL	*L. nimipressuralis*; 84.39%; P_134349910.1
EL-524	E5283_02605	BluR	62.03	BLUF-EAL	*Leclercia* sp.; 90.39%; WP_048027580.1
EL-769	E5283_03840	-	-	Unknown-HTH-LuxR-EAL	*Leclercia* sp.; 90.39%; WP_048027580.1
EL-905	E5283_04505	BluR	44.50	BLUF-EAL	*L. nimipressuralis*; 87.87%; WP_134350370.1
EL-1383	E5283_06890	YoaD	68.61	CSS-EAL	*Klebsiella* sp.; 64.75%; WP049840643.1
EL-1650	E5283_08250	-	-	Unknown-EAL	*L. nimipressuralis*; 87.87%; WP_134350370.1
EL-1742	E5283_08705	Rtn	60.51	CSS-EAL	*Mesorhizobium* sp.; 90%; TIN52519.1
EL-2984	E5283_15070	YhjH	72.73	EAL	*Lelliottila aquatilis*; 81.95%; WP_103946061.1
EL-3490	E5283_17765	YjcC	63.13	CSS-EAL	*L. nimipressuralis*; 95.59%; WP_134347305.1
EL-4205	E5283_21415	YlaB	59.69	CSS-EAL	*Citrobacter* sp.; 61.97%; WP121585463.1
EL-4271	E5283_21750	-	-	EAL	*Mesorhizobium* sp.; 90%; TIN52519.1
**GGDEF-EAL domain proteins**					
EL-640	E5283_03185	-	-	CHASE4-GGDEF-EAL	*Lelliottia* sp.; 82.64%; WP059178784.1
EL-1064	E5283_05280	-	-	Unknown-GGDEF-EAL	*Citrobacter* sp.; 58.12%; WP086551269.1
EL-1108	E5283_05495	YciR	78.78	PAS-GGDEF-EAL	*L. nimipressuralis;* 97.44%; TFB20523.1
EL-1647	E5283_08235	YegE	75.32	MASE1-PAS-PAS_PAS-GGDEF-xEAL	*Mesorhizobium* sp.; 92.79%; TIM58473.1
EL-1899	E5283_09475	YfeA	58.25	MASE1-xGGDEF-EAL	*L. nimipressuralis*; 80.07%; OIR50653.1
EL-1965	E5283_09845	YfgF	62.81	MASE1-xGGDEF-EAL	*L. nimipressuralis*; 90.43%; WP_134347661.1
EL-2734	E5283_13795	CsrD	77.55	GAPES4-xGGDEF-xEAL	*L. nimipressuralis*; 80.07%; OIR50653.1
EL-2748	E5283_13865	-	-	MHYT-GGDEF-EAL	*Kosakonia* sp.; 79.94%; SEL52885.1
EL-2988	E5283_15090	YhjK	78.15	GAPES3-HAMP-xGGDEF-EAL	*L. nimipressuralis*; 90.43%; WP_134347661.1
EL-4362	E5283_22180	-	-	Unknown-GGDEF-EAL	*Citrobacter* sp.; 65.63%; WP003836475.1
**HD-GYP domain proteins**					
EL-1478	E5283_07365	-	-	SBP_bac_3-SBP_bac_3-HD-GYP	*Lelliottia* sp.; 72.47%; WP103949684.1

^1^ locus tags of *E. ludwigii* CEB04 based on publicy available NCBI annotation; ^2^ indicates absence of homologous protein in *E. coli* MG1655; ^3^ % amino acid identity to *E. coli* MG1655 homologue over the entire length of the protein.

## Supplementary Materials

The following are available online at https://www.mdpi.com/2076-2607/8/4/522/s1.

## Data

The sequencing data were deposited to GeneBank under bioproject PRJNA531388 with INSDC (International Nucleotide Sequence Database Collaboration) number CP039741. *E. ludwigii* CEB04 was deposited in the Culture Collection University of Gothenburg (CCUG) collection, a Swedish depository for clinically relevant bacteria.
