# Supplementary material for: Draft Genome Sequence of the Urinary Catheter Isolate Enterobacter ludwigii CEB04 with High Biofilm Forming Capacity
Source: Microorganisms. 2020 Apr 5;8(4):522. doi: 10.3390/microorganisms8040522 (PMC7232144; doi:10.3390/microorganisms8040522)

microorganisms

MDPI

Supplementary data

Draft Genome Sequence of the Urinary Catheter Isolate *Enterobacter ludwigii* CEB04 with High Biofilm Forming Capacity

Sulman Shafeeq [1], Xiaoda Wang [1], Heinrich Lünsdorf [2], Annelie Brauner [1,3] and Ute Römling [1,*]

[1] Department of Microbiology, Tumor and Cell Biology, Karolinska Institutet, Stockholm SE-171 65, Sweden; sulman.shafeeq@ki.se (S.S.); wangxiaoda@hotmail.com (X.W.); Annelie.Brauner@ki.se (A.B.)
[2] Helmholtz Center for Infection Research, Braunschweig, DE-38124, Germany; hlunsdorf@web.de
[3] Clinical Microbiology, Karolinska University Hospital, Stockholm SE-171 76, Sweden
* Correspondence: ute.romling@ki.se

microorganisms

MDPI

Figure S1: Alignment of GGDEF domains in *E. ludwigii* CEB04. EL= *E. ludwigii*, ST= *S. typhimurium* and CC= *Caulobacter crescentus*.

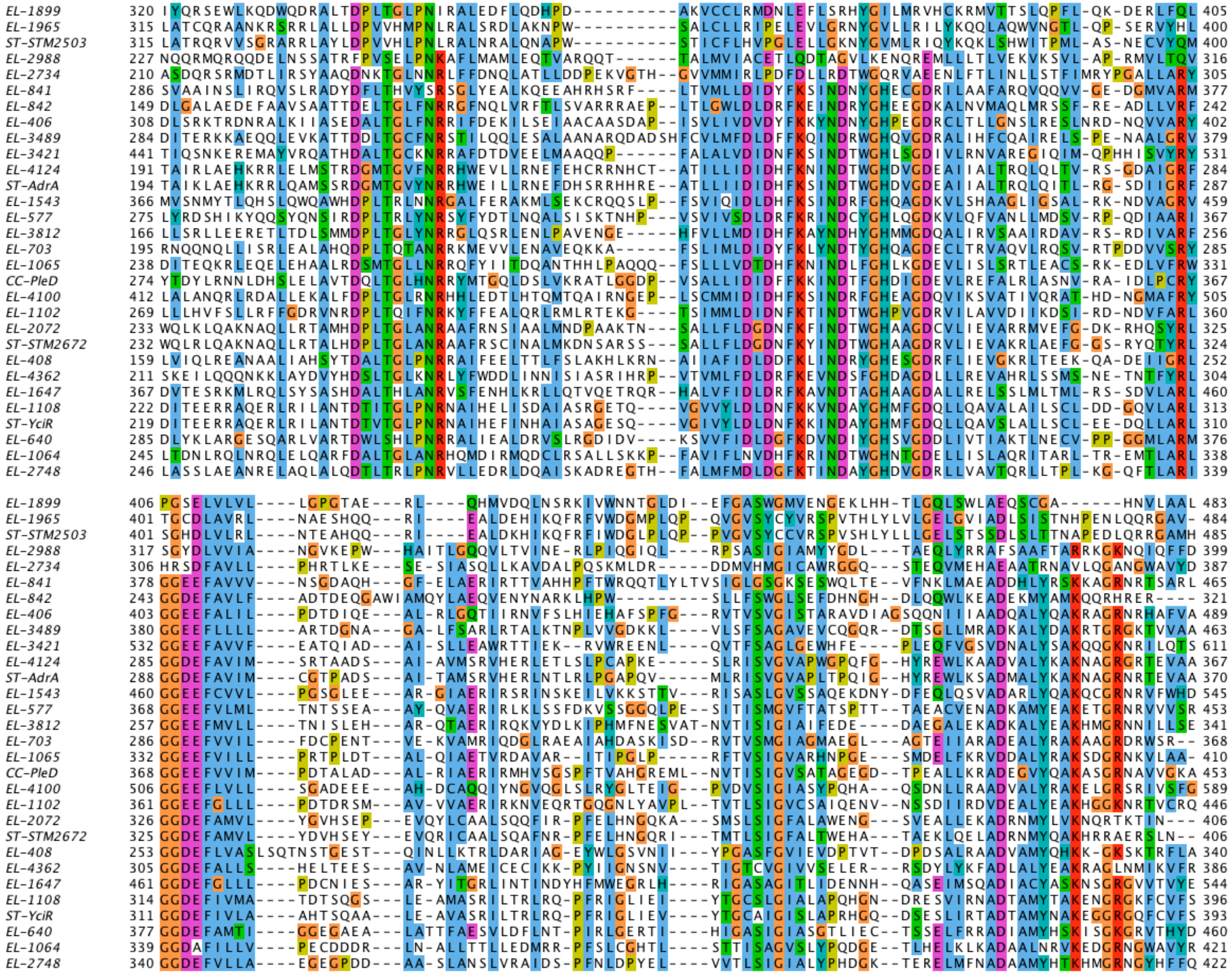

microorganisms

MDPI

Figure S2: Alignment of EAL domains in *E. ludwigii* CEB04. EL= *E. ludwigii*, ST= *S. typhimurium* and PA= *Pseudomona aeruginosa*.

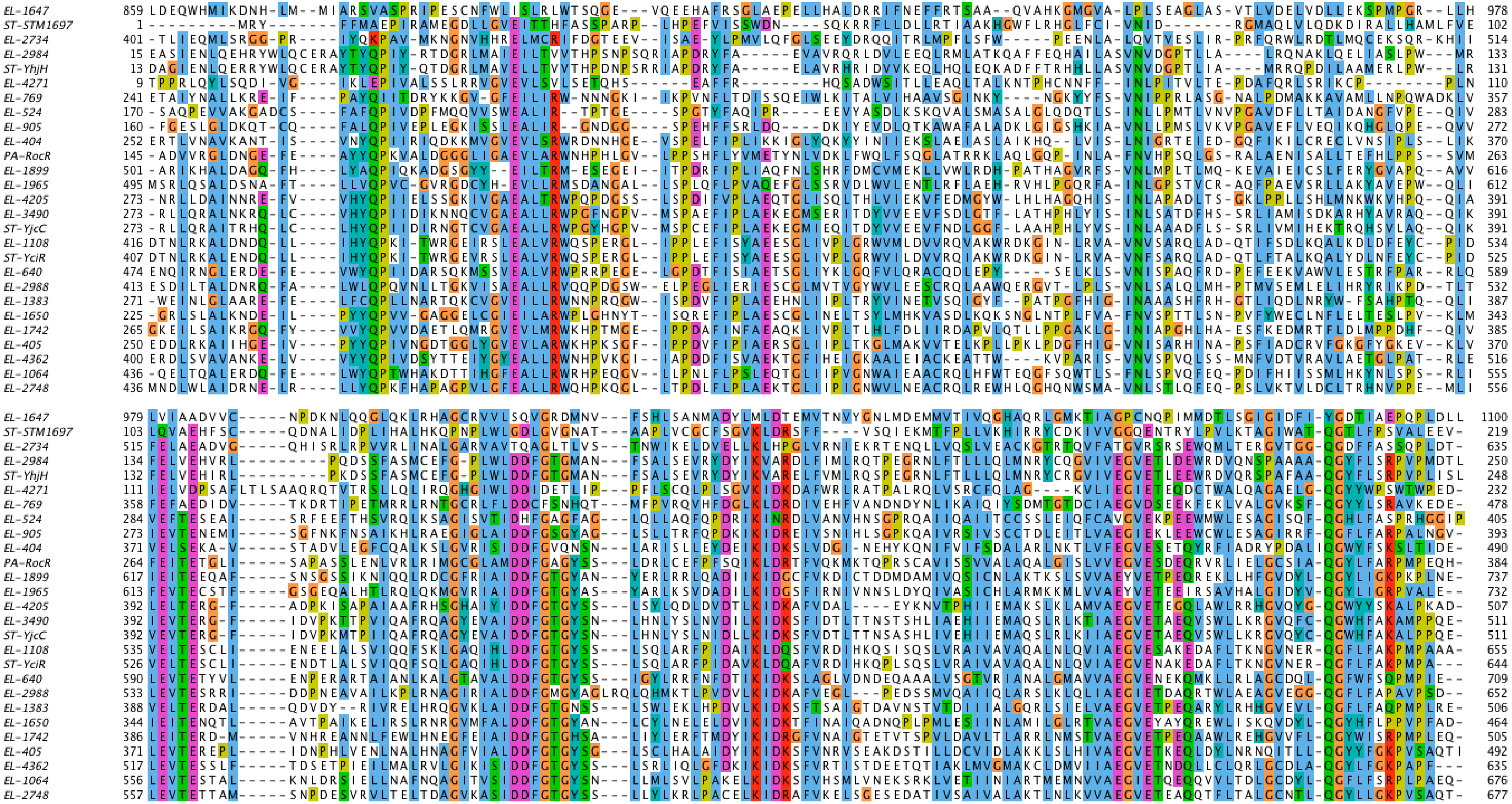

Supplement: Supplementary file 1 [file microorganisms-08-00522-s001.pdf]
